# Protocol for a cross-sectional study on factors affecting health-related quality of life among Afghan refugees in Pakistan

**DOI:** 10.12688/f1000research.73005.2

**Published:** 2024-10-14

**Authors:** Atta Ur Rehman, Rubeena Zakar, Muhammad Zakria Zakar, Ume Hani, Florian Fischer

**Affiliations:** 1 Institute of Social and Cultural Studies, University of the Punjab, Lahore, Pakistan; 2 University of Okara, Okara, Pakistan; 3 Holy Family Hospital, Rawalpindi Medical University, Rawalpindi, Pakistan; 4 Institute of Gerontological Health Services and Nursing Research, Ravensburg-Weingarten University of Applied Sciences, Weingarten, Germany; 5 Institute of Public Health, Charité-Universitätsmedizin Berlin, Berlin, Germany

**Keywords:** Afghanistan, refugees, migration, health, quality of life

## Abstract

**Background:**

Pakistan served as a host for more than 1.4 million Afghan refugees for more than 40 years. Access to health care is the most important issue faced by refugees, because they might be at a higher risk for certain diseases. This risk can be attributed to a lack of awareness of health care facilities, health beliefs, inadequate hygiene, cultural differences, and malnutrition. Health of individuals is closely associated with their quality of life. Quality of life over the whole lifespan is pivotal to overall life satisfaction. It includes physical wellbeing, mental health, education, occupation, income, personal safety, as well as (religious) freedom. Until now, the health status of Afghan refugees has never been comprehensively investigated in Pakistan. Therefore, an assessment in this regard is needed to explore their health-related quality of life, for securing their human right to health.

**Methods:**

A cross-sectional study has been designed to describe and explain the health-related quality of life of Afghan refugees in Pakistan. Multistage cluster sampling was applied for selection of study participants. The number of respondents from two regions in Pakistan was drawn through a proportionate sampling technique. A quantitative research method using pre-validated questionnaires was used for data collection. The questionnaire included items to assess well-being, mental health, health literacy, and factors affecting health and health care. Descriptive analysis was used, whereas inferential statistical tests (binary logistic regression model) was also performed. The study received ethically permission by the Advanced Studies and Research Board of the University of the Punjab, Lahore, Pakistan.

**Discussion:**

The assessment of Afghan refugee’s quality of life in Pakistan should lead to recommendations disseminated to public and health care officials. This evidence is needed for policymaking related to adequate measures for improving health conditions of Afghan refugees in Pakistan.

## Abbreviations

AAHLS: All Aspects of Health Literacy Scale

QOL: Quality of life

SPSS: Statistical Package for the Social Sciences

UNHCR: United Nations High Commissioner for Refugees

WHO: World Health Organization

## Introduction

The World Health Organization (WHO) advocated health as a fundamental human right in its constitution of 1946. The availability of health care facilities to all individuals – irrespective of gender, religion, race, political, economic and social conditions – are essential to attain this right. ^1^ However, war is considered as a serious threat to this human right. ^2^ War may lead to displacement and refugees are usually most vulnerable in host countries and, therefore, are at high risk of developing certain diseases. ^3^ Refugees might face exploitation, prejudice and violence during travelling and stay in host countries which may negatively impact on their health. ^4^

For about 40 years, Pakistan served as a host for more than 1.4 million Afghan refugees. ^5^ Health care is one of the most important issues faced by refugees during migration. Health is a state of complete physical, mental and social well-being and not merely the absence of disease or infirmity. ^6^ Health of individuals living in a particular society is dependent on their quality of life. According to the WHO, quality of life is defined as “the individual’s perception of their position in life in the context of the culture and value systems in which they live and in relation to their goals”. ^7^ It describes well-being of individuals and society considering the positive and negative features of life. Quality of life across the whole span is pivotal to satisfaction in life. That includes physical well-being, mental health, academic achievements, job, income, personal safety, and (religious) freedom. ^8^ Health disparities are prevalent in almost all societies across the globe. Health inequalities and financial constrains may result in psychological health problems. ^9^

Refugee’s health is mostly dependent on living conditions and health facilities in host countries. Refugees might be at a higher risk for getting certain diseases because of lack of awareness of health facilities, health beliefs, inadequate hygiene, cultural differences, and malnutrition. Their health problems and needs may vary over time. ^10^ At the time of arrival, refugees might face health problems related to injuries, gastrointestinal disorders, infectious diseases, cardiovascular disorders, hypothermia, skin diseases, mental health disorders, malnutrition and women’s health needs during pregnancy and delivery. After resettlement, refugees are at risk of mental health problems, communicable diseases, and non-communicable diseases. Particularly women and children are vulnerable populations. They may experience exposure to violence during their stay in refugee camps from local population as well as from other refugees. ^11^

The pattern of migration has changed over time, but the factors affecting the quality of life and psychological well-being remain the same. The large influx of Afghan refugees has affected neighbouring countries such as Pakistan. Afghan’s presence in the labour market directly affects their quality of life, because they are often informally employed without access to social protection. ^12^ Although Afghan refugees are present in Pakistan for more than four decades, there is a lack of research to assess their quality of life. The quality of life of refugees affects not only their ability to fully participate in society but also acts as a barrier to learn new skills. Health-related quality of life is influenced by individual factors (age, sex, genes), lifestyle factors (socioeconomic, cultural, linguistic barriers and substance abuse), ^13^ living conditions (access to clean water, sanitation and housing), working conditions (access to work, job), social and community factors (discrimination, social inclusion) and governance (documentation). ^8^ Knowledge about the health care system as well as health literacy are also important factors. ^14^

Mental health and psychological well-being are the basis for social functioning of any human being. Health care professionals used the terminology well-being to relate health-related quality of life and mental health. ^15^ Overall, major depression was found in 30.8% of refugees, whereas post-traumatic stress disorder prevalence rate was 30.6%. ^16^ Investigations on war-influenced youths have identified changes in psychological well-being on social encounters. Trauma memories, mental health, and resilience were investigated by Panter-Brick *et al.* ^17^ in Afghan youths (11–16 years old) with their caregivers in Kabul (Afghanistan) and Peshawar (Pakistan). This study provided evidence on the association between posttraumatic distress and depression in Afghan youth. Furthermore, it showed that posttraumatic distress was less frequently observed in males as compared to females. ^17^

Badshah *et al.* ^18^ investigated maternal risk factors in Pakistani mothers compared to Afghan-refugees in Peshawar, Khyber Pakhtunkhwa, Pakistan. The study showed a 2.6 times higher likelihood for low birth weight of neonates among the Afghan refugees. The reasons for low birth weight include living in tribal areas, no access to fresh water, low income, abortion/miscarriages, unregistered pregnancies, short inter-pregnancy intervals, and old age. However, there is a need for further research to assess the factors impacting on maternal health in Afghan refugees to overcome their health risks. ^18^ This assessment is needed to explore the determinants of health-related quality of life, health beliefs, and the current health status of refugees in Punjab and Khyber Pakhtunkhwa, Pakistan.

The literature from developed countries, including the European Union, United States, and Australia focuses on various aspects of refugee health assessment, such as health-related quality of life, mental health screening, and access to healthcare, food insecurity, and language proficiency in the host country. However, the specific type of health assessment conducted may vary depending on the country’s policies and the author’s research focus as presented in Table 1. ^19^^–^^42^

**Table 1.** Summary of review of literature from developed countries.

Author	Year & Country	Research Method & Sample size	Research Aim	Main Outcomes
Solberg *et al*	2022 Sweden	Quantitative 10,000	Socio demographics relationship with health-associated quality of life was explored	Refugee targeted population had low scores in social & peer support and psychological health in comparison to the European population
Matsangos *et al*	2022 Europe	Review of literature	Review of literature on policy and practical recommendations to enhance Afghan refugees’ existing health status	The most prevalent diagnoses in terms of psychological disorders were post-traumatic stress disorder and depression
Borho *et al*	2022 Germany	Quantitative 116	Psychological health assessment of Syrian migrants in Germany using the Refugee Health Screener versions 13 and 15	Refugee health screener Versions 13 and 15 both detected psychological distress in 57% and 66% of subjects, respectively
Feinberg *et al*	2022 USA	Quantitative 136	To assess the relationship between refugee general health, linguistic ability, educational achievement, and time consumed in the host country	The amount of time spent in the host country was modestly associated with general health. Language ability and health literacy were both highly predictive of overall health
Riggs *et al*	2020 Australia	Qualitative 64	To determine how easily accessible prenatal and postnatal healthcare information was to Afghan households and healthcare professionals	The inadequate information provided was likely the root cause of Afghan couples' poor health literacy as well as the poor neonatal and maternal health outcomes
Husby *et al*	2020 Denmark	Mixed Quantitative and Qualitative 92	To investigate intervention acceptance by using WHO-5	The marked increase in the WHO-5 indicates a beneficial effect on depressed mood and general wellbeing
Kohlenberger *et al*	2019 Austrian	Quantitative 500	To assess psychological well-being and their perceptions of medical services	Two out of ten men and four out of ten women refugees reported experiencing underserved healthcare needs
Im *et al*	2018 USA	Qualitative 25	To assess the level of critical health literacy among Congolese and Afghan immigrants to the United States	This research highlighted the significance of health education confirming social awareness in a group environment for the development of social support networks
Yaser *et al*	2017 Australia	Qualitative 150	To investigate the level of mental health literacy among Afghan refugees	Thematic assessment of qualitative conversations revealed that several respondents categorically classified trauma as a result of pre-arrival experience with conflict and harassment
Kaltenbach *et al*	2017 Germany	Quantitative 86	To identify psychological health issues in Germany by using a Refugee health screener (15)	The refugee health screener 15 revealed existing psychological problems in 52 % of the refugee population
Slewa-Younan *et al*	2017 Australia	Quantitative 150	To evaluate the mental health status with related help-seeking behavior of Afghan refugees in Adelaide, Australia	The results of the study revealed that 14.7% of participants had symptoms of depression. General practitioners were contacted mostly for help-seeking
Khakpour *et al*	2017 Switzer land	Quantitative 25	To examine the effect of cultural and socioeconomic predictors on food insecurity among Afghan refugee households in Switzerland	The outcomes of the survey indicated that because of economic and cultural differences, severe food insecurity exists among Afghan asylum-seeker families. More than 70% of the Asylum-seekers were food insecure
Stempel *et al*	2017 USA	Quantitative 259	This cross-sectional survey explored the influence of apparent discrimination on the psychological wellness of Afghan refugees	The findings indicated that discrimination was a major stress contributor for Afghan refugees, which may aggravate stress related to post-migration resettlement factors
Shabaik *et al*	2016 USA	Review of literature 26	This investigation aimed to thoroughly review existing literature that highlights those aspects of the health-sickness transition condition that refugees experience during migration	Qualitative data indicated the role of gender, family issues, and aging as factors in the health-sickness transition state whereas quantitative data described high lipid profile, the incidence of cancer, and the increased possibility of psychological complications as determinants of health-sickness transition condition
Alemi *et al*	2015 USA	Quantitative 135	To identify the mental distress indicators among Afghan refugees in California State, USA	The outcome of this investigation highlighted the social determining factors of mental distress among Afghan refugees in the USA and proposed further assessment for better mental health outcomes
Wångdahl *et al*	2015 Sweden	Quantitative 360	To determine whether interactions between refugees and healthcare professionals during their medical evaluation for refugees were influenced by their level of health literacy	The 36% of participants reported inadequate communication, 55% reported little understanding of health, 41% reported limited new information, and 26% reported receiving some assistance
Wångdahl *et al*	2014 Sweden	Quantitative 455	To assess functional and comprehensive health literacy rates in refugees	Most of the refugees who participated in the research had either insufficient or deficient functional and comprehensive health literacy

## Methods

### Hypotheses and aims

Firstly, we consider that individual and lifestyle factors are associated with health-related quality of life among Afghan refugees in Pakistan. Secondly, we presume that there are differences in health-related quality of life due to living and working conditions among Afghan refugees in Pakistan. Thirdly, we expect that there is an association between social and community factors affecting health-related quality of life among Afghan refugees in Pakistan.

The main objectives of the study are:

1. to determine the health-related quality of life among Afghan refugees in Punjab and Khyber Pakhtunkhwa, Pakistan,
2. to examine the factors affecting health-associated quality of life and study their association among Afghan refugees in Punjab and Khyber Pakhtunkhwa, Pakistan,

The results should support to suggest measures to improve the health status of Afghan refugees in Punjab and Khyber Pakhtunkhwa, Pakistan.

### Study design

The investigation design was cross-sectional. The sampling frame for the current investigation was refugee’s population in Khyber Pakhtunkhwa and Punjab, Pakistan. based on data from the United Nations High Commissioner for Refugees (UNHCR). ^43^ Khyber Pakhtunkhwa is accommodating more than 58% of the Afghan refugee population. Punjab is the largest local educated population province with maximum human development index in the country. Thus, both provinces qualify as a favorable setting for Afghan refugees’ survey. We used validated instruments to collect information from Afghan refugees.

### Sampling technique

Multistage sampling was used to collect on-site data. In the first stage, clusters of Afghan refugees (District) were selected from each province by online data available. Clusters were selected on the basis of both sexes’ participation in the pilot survey, because in some districts husbands did not allow their wives to take part in the study due to sociocultural factors. In the second stage, the number of Afghan participants were estimated from each district through proportionate sampling technique based on the population in each district. Finally, in the third stage, the calculated sample size was completed systematically due to non-data (refugee list) sharing policy of organizations working with the refugees. From the first ten refugee household’s, the second was selected through random number generator. The desired sample size was achieved through visiting the households at a regular interval. The Afghan Proof of Registration (PoR) card was observed as a proof of their refugee status in the resident area.

### Sample size calculation

Sampling formula for known population was used:

$$\begin{array}{ll} \text{Size}\;\text{of}\;\text{Sample n} & =\;\text{N}/\left[1+\text{N}{\left(e\right)}^{2}\right]\\ & =35,082/\left[1+35,082{\left(.03\right)}^{2}\right]\\ & =1,077 \end{array}$$

N is total population of refugee families 35,082 and e ± 3% level of precision (sampling error). The chances of rejection were assumed as 10%. Therefore, the sample size was estimated at 1,185.

Proportionate random sampling technique was used to calculate actual sample size. The formula used to calculate the number of sample families in each province is:

Formula (Sampling) = n/N * 100

Table 2 illustrates the selection of Afghan refugees within the provinces. ^5, 44, 45^

**Table 2.** Selection of sample size among the provinces.

Province (City)	Total families	Sample size	Proportion
**Khyber Pakhtunkhwa**	**32674**	**1104**	**0.93**
Haripur	11731	396	0.36
Mardan	2226	75	0.07
Peshawar	11662	394	0.36
Nowshera	7055	239	0.21
**Punjab**	**2408**	**81**	**0.07**
Kot Chandana Mianwali	2408	81	0.07
**Total families**	**35082**	**1185**	**1.0**

### Inclusion and exclusion criteria

The refugee families accessible in the study area during the time of data collection willing to participate were included in the study. Refugees with inability to understand consent procedures and to reply a questionnaire were excluded from the investigation. The objectives of the study were clearly explained to the families before the questionnaires were administered and written informed consent was obtained. Sampled families were guaranteed confidentiality and anonymity of the data throughout the study.

### Tools of data collection

Data was collected by using pre-validated questionnaires ( Table 3). Quality of life was assessed with the World Health Organization Quality-of-Life Scale (WHOQOL-BREF) by applying standardized age and gender sampling quota. ^46^ According to sampling quota, 50% of the participants must be female and age-wise 50% of the participants must be older than 45 years of age. Psychological well-being was assessed by WHO-Wellbeing index ^47^ and mental health by the Refugee Health Screener. ^48^ The All Aspects of Health Literacy Scale (AAHLS) was used to measure health literacy. ^49^ Questionnaires related to factors affecting health and health care were developed from Syrian Refugee Health Access Survey in Jordan and Lebanon according to local needs of the population. ^50– 52^ The existing internationally used instruments version with documented validity and reliability of English, Urdu and Afghan national Dari language was used. Permissions to use the tools were given by the stakeholders/authors. Afghan national bilingual research assistants were engaged for the data collection and linguistic interpretations of the Afghan national language according to the guidelines of WHO. Forward and backward translations were conducted under supervision of the principal investigator. The paper-based research questionnaires were administered to male respondents by Afghan national bilingual research assistants under the supervision of the first author and to female respondents by the fourth author with the help of research assistants in the research area. A team of two principal investigators and four trained research assistants supported the participants to fill in the questionnaires where needed. The research assistants were trained for two weeks before the field work.

**Table 3.** Measurement instruments of research variables.

Variables	Measurement tool
Quality of life	WHOQOL-BREF
Well-being	WHO-Wellbeing index
Mental health	Refugee Health Screener
Health literacy	All Aspects of Health Literacy Scale
Factors affecting health and health care	Syrian Refugee Health Access Survey in Jordan and Lebanon

### Data analysis

The data of the filled-out forms has been entered in SPSS version 24 for data analysis. Descriptive analysis includes calculation of frequencies and percentages, whereas inferential statistical tests were applied to measure the level of association between variables. ^53^ Logistic regression was performed to examine the relationship between variables at a 0.05 level of significance. The power 1-β of the test is 0.80 at 95% confidence level. The relevant differences should be observed by odds ratios.

The data of the current investigation will be shared with scientific journals and associated data repositories. The data was coded and sub-coded to ensure that participant identification may not be revealed. The analysis was carried out by the principal investigator who is trained. The supervisors and team members also supported and guided during the analysis.

### Sources of bias

We tried to remove selection bias from the project by choosing a representative sample by utilizing WHO quality of life standard age and gender sampling quota. According to this, 50% of the participants must be female and age-wise 50% of the participants must be older than 45 years. As the final sample was collected by applying systematic random sampling, we make sure that the randomization of the participants in terms of age and gender must be followed to complete the sample size from the selected cluster. Although the refusal rate was quite high in the refugee population, we tried to include at least 50% of the respondents that responded to any survey for the first time. Appropriate probability sampling techniques and internationally accepted questionnaires are used to avoid recall bias. Interviewees were given sufficient time for recall of memory. The sample size calculated was 1,185 so that maximum responses should be available to conclude the results. Specific inclusion and exclusion criteria were established at the design stage so that our outcomes are correctly identified.

### Ethical considerations

The study is ethically permitted by the Advanced Studies and Research Board of the University of the Punjab, Lahore. Written informed consent for voluntary participation was obtained from respondents. The objectives of the study had been clearly explained to the participating families before the questionnaires were administered and written informed consent was obtained. Sampled families were guaranteed voluntary participation, confidentiality, and anonymity of the data throughout the study.

## Plans for dissemination of the study outcome

The results are going to be disseminated to the public by open defence and health care officials via outcome sharing, as this evidence is needed for policymaking related to adequate measures for improving health conditions of Afghan refugees in Pakistan. This investigation results will be submitted in the form of draft to the University of the Punjab, Lahore, Pakistan and Higher Education commission library, Islamabad for the record and guidance for the future investigations. The research will be submitted in the form of research papers to international journals.

## Study status

Data collection was initiated in March 2020 and completed in May 2021. Currently, the data is analyzed. A first manuscript draft will be finished by October 2021.

## Discussion

The Pakistani health care system met a number of public health challenges over the past decades. Afghan refugees are a significant population group which has to be served by provincial health facilities. In the 1980s and 1990s, the government of Pakistan focused mainly on addressing basic health care needs of refugees related to epidemics like malaria. More recently, the attention shifted to providing antenatal care and child health immunization coverage among refugees in Pakistan. ^54^ The strong focus on infectious diseases relates to a study by UNHCR in 2012, which emphasizes that the main health problems faced by refugees were skin diseases, typhoid, malaria, diarrhoea, measles, dysentery, hepatitis C, thalassemia, cholera and tuberculosis. ^55^ Afghan refugees experience several health risks which may increase the risk of infectious diseases. For that reason, Afghan refugees contributed to the spread of polio in Pakistan. ^56^ However, their health status has never been comprehensively evaluated.

This study will provide an overview of the health-related quality of life of Afghan refugees in Punjab and Khyber Pakhtunkhwa. A comprehensive approach using pre-validated questionnaires was used for assessment. The outcomes of families in different provinces and clusters will be compared for living conditions and social inclusion in their host areas.

## Limitations

Study limitations are that participants with inability to response to consent procedures and questionnaires were not included in the investigation. As for all epidemiological surveys, the inclusion of marginalised groups, such as refugees, is a major challenge for all kinds of surveys, particularly for health surveys. Although a random sampling technique was applied, various sampling procedures have shown limited success in migrant health research. Despite these limitations, the findings of the study will provide information on the current health status of Afghan refugees. There is a need for research in this regard to provide better health care facilities to refugees.

## Data availability

No data are associated with this article.

## References

[1] Lougarre C : Using the Right to Health to Promote Universal Health Coverage: A Better Tool for Protecting Non-Nationals’ Access to Affordable Health Care? *Health Hum Rights.* 2016;18:35–48. 28559675 PMC5395007

[2] Yusuf S Anand S MacQueen G : Can medicine prevent war? Imaginative thinking shows that it might. *BMJ.* 1998;317:1669–1670. 10.1136/bmj.317.7174.1669 9857115 PMC1114475

[3] Langlois EV Haines A Tomson G : Refugees: towards better access to health-care services. *Lancet.* 2016;387:319–321. 10.1016/S0140-6736(16)00101-X 26842434 PMC5603273

[4] Abbas M Aloudat T Bartolomei J : Migrant and refugee populations: a public health and policy perspective on a continuing global crisis. *Antimicrob Resist Infect Control.* 2018;7:113. 10.1186/s13756-018-0403-4 30250735 PMC6146746

[5] UNHCR: *Operational Portal Refugee Situations.* Brussels: United Nations High Commissioner for Refugees;2020.

[6] Kuhn S Rieger UM : Health is a state of complete physical, mental and social well-being and not merely absence of disease or infirmity. *Surg Obes Relat Dis.* 2017;13:887. 10.1016/j.soard.2017.01.046 28389194

[7] Gholami A Jahromi LM Zarei E : Application of WHOQOL-BREF in Measuring Quality of Life in Health-Care Staff. *Int J Prev Med.* 2013;4:809–817. 24049600 PMC3775221

[8] Martinez-Martin P Prieto-Flores ME Forjaz MJ : Components and determinants of quality of life in community-dwelling older adults. *Eur J Ageing.* 2012;9:255–263. 10.1007/s10433-012-0232-x 28804425 PMC5547413

[9] Magklara K Skapinakis P Niakas D : Socioeconomic inequalities in general and psychological health among adolescents: a cross-sectional study in senior high schools in Greece. *Int J Equity Health.* 2010;9:3. 10.1186/1475-9276-9-3 20181002 PMC2837664

[10] Pavli A Maltezou H : Health problems of newly arrived migrants and refugees in Europe. *J Travel Med.* 2017;24. 10.1093/jtm/tax016 28426115

[11] Daynes L : The health impacts of the refugee crisis: a medical charity perspective. *Clin Med.* 2016;16:437–440. 10.7861/clinmedicine.16-5-437 27697805 PMC6297302

[12] Morgan WA : Experience of a clinic for Afghan refugees in Pakistan. *West J Med.* 1988;149:234–238. 3247740 PMC1026399

[13] Braveman P Gottlieb L : The social determinants of health: it's time to consider the causes of the causes. *Public Health Rep.* 2014;129(Suppl. 2):19–31. 10.1177/00333549141291S206 24385661 PMC3863696

[14] Wangdahl J Lytsy P Martensson L : Health literacy and refugees' experiences of the health examination for asylum seekers - a Swedish cross-sectional study. *BMC Public Health.* 2015;15:1162. 10.1186/s12889-015-2513-8 26596793 PMC4657287

[15] Soutter AK O'Steen B Gilmore A : The student well-being model: a conceptual framework for the development of student well-being indicators. *Int J Adolesc Youth.* 2014;19:496–520. 10.1080/02673843.2012.754362

[16] Steel Z Chey T Silove D : Association of torture and other potentially traumatic events with mental health outcomes among populations exposed to mass conflict and displacement: a systematic review and meta-analysis. *JAMA.* 2009;302:537–549. 10.1001/jama.2009.1132 19654388

[17] Panter-Brick C Grimon MP Kalin M : Trauma memories, mental health, and resilience: a prospective study of Afghan youth. *J Child Psychol Psychiatry.* 2015;56:814–825. 10.1111/jcpp.12350 25384553

[18] Badshah S Mason L McKelvie K : Maternal risk factors in Afghan-refugees compared to Pakistani mothers in Peshawar, NWFP Pakistan. *J Pak Med Assoc.* 2011;61:161–164. 21375167

[19] Abernathy JR Greenberg BG Wells HB : Smoking as an independent variable in a multiple regression analysis upon birth weight and gestation. *Am J Public Health Nations Health.* 1966;56:626–633. 10.2105/AJPH.56.4.626 5949658 PMC1256998

[20] Alemi Q James S Siddiq H : Correlates and Predictors of Psychological Distress among Afghan Refugees in San Diego County. *Int J Cult Ment Health.* 2015;8:274–288. 10.1080/17542863.2015.1006647 26543500 PMC4629859

[21] Borho A Morawa E Erim Y : Mental Health Screening of Syrian Refugees in Germany: The Refugee Health Screener. *Z Psychosom Med Psychother.* 2022;68:269–282. 10.13109/zptm.2022.68.oa1 35380103

[22] Flannelly LT Flannelly KJ Jankowski KR : Independent, dependent, and other variables in healthcare and chaplaincy research. *J Health Care Chaplain.* 2014;20:161–170. 10.1080/08854726.2014.959374 25255148

[23] Feinberg I O'Connor MH Owen-Smith A : The Relationship Between Refugee Health Status and Language, Literacy, and Time Spent in the United States. *Health Lit Res Pract.* 2020;4:e230–e236. 10.3928/24748307-20201109-01 33313933 PMC7751446

[24] Grønning K Espnes GA Nguyen C : Psychological distress in elderly people is associated with diet, wellbeing, health status, social support and physical functioning- a HUNT3 study. *BMC Geriatr.* 2018;18:205. 10.1186/s12877-018-0891-3 30180808 PMC6123986

[25] Husby SR Carlsson J : Mathilde Scotte Jensen A, Glahder Lindberg L, Sonne C: Prevention of trauma-related mental health problems among refugees: A mixed-methods evaluation of the MindSpring group programme in Denmark. *J Community Psychol.* 2020;48:1028–1039. 10.1002/jcop.22323 32027393

[26] Im H Swan LE : Qualitative exploration of critical health literacy among Afghan and Congolese refugees resettled in the USA. *Health Edu J.* 2019;78:38–50. 10.1177/0017896918785932

[27] Kaltenbach E Härdtner E Hermenau K : Efficient identification of mental health problems in refugees in Germany: the Refugee Health Screener. *Eur J Psychotraumatol.* 2017;8:1389205. 10.1080/20008198.2017.1389205 29163869 PMC5687797

[28] Kohlenberger J Buber-Ennser I Rengs B : Barriers to health care access and service utilization of refugees in Austria: Evidence from a cross-sectional survey. *Health Policy.* 2019;123:833–839. 10.1016/j.healthpol.2019.01.014 30878171

[29] Khakpour M Sadeghi L Jenzer H : The Impact of Soci-economic and Cultural Factors on Refugee Households' Food Insecurity: a Snapshot of the Food security Status of Afghan Refugees in Switzerland. *FASEB J.* 2018;31. 10.1096/fasebj.31.1_supplement.791.13

[30] Matsangos M Ziaka L Exadaktylos AK : Health Status of Afghan Refugees in Europe: Policy and Practice Implications for an Optimised Healthcare. *Int J Environ Res Public Health.* 2022;19:9157. 10.3390/ijerph19159157 35954518 PMC9368211

[31] Atchi F : Effet de l’inclusion financière sur la pauvreté multidimensionnelle au Togo. *Revue Internationale des Économistes de Langue Française.* 2022;7:197–214. 10.18559/rielf.2022.1.11

[32] Purba J Budiono S : *Availability of Electricity, Clean Water and Sanitation towards Economic Growth in Indonesia with 500 Regencies and Cities.* 2020.

[33] Riggs E Yelland J Szwarc J : Afghan families and health professionals' access to health information during and after pregnancy. *Women Birth.* 2020;33:e209–e215. 10.1016/j.wombi.2019.04.008 31097412

[34] Salleh M Mohi Z Nordin N : The Impact of Language Barriers and Discrimination Issues on Work Productivity of Foreign Workers. *International Journal of Academic Research in Business and Social Sciences* 2021;11:11. 10.6007/IJARBSS/v11-i16/11215

[35] Shabaik HS Lee E : *Health-illness transition of first generation refugees: A review of literature on Afghan refugees.* 2016.

[36] Slewa-Younan S Yaser A Guajardo MGU : The mental health and help-seeking behaviour of resettled Afghan refugees in Australia. *Int J Ment Health Syst.* 2017;11:49. 10.1186/s13033-017-0157-z 28855961 PMC5571658

[37] Stempel C Sami N Koga PM : Gendered sources of distress and resilience among Afghan refugees in Northern California: a cross-sectional study. *Int J Environ Res Public Health.* 2017;14:25. 10.3390/ijerph14010025 PMC5295276 28036054

[38] Solberg Ø Sengoelge M Johnson-Singh CM : Health-related quality of life in refugee minors from Syria, Iraq and Afghanistan resettled in Sweden: a nation-wide, cross-sectional study. *Soc Psychiatry Psychiatr Epidemiol.* 2021;57:255–266. 10.1007/s00127-021-02050-8 33754158 PMC8784357

[39] Vogel CE Molinari V Andel R : Self-rated health and mental health among older incarcerated males. *Aging Ment Health.* 2021;25:2100–2108. 10.1080/13607863.2020.1795621 32698603 PMC7855989

[40] Wångdahl J Lytsy P Mårtensson L : Health literacy among refugees in Sweden–a cross-sectional study. *BMC Public Health.* 2014;14:1030. 10.1186/1471-2458-14-1030 25278109 PMC4195944

[41] Wångdahl J Lytsy P Mårtensson L : Health literacy and refugees’ experiences of the health examination for asylum seekers–a Swedish cross-sectional study. *BMC Public Health.* 2015;15:1162. 10.1186/s12889-015-2513-8 26596793 PMC4657287

[42] Yaser A : *Mental health literacy of resettled Afghan refugees in Australia: an exploration of how resettled refugees understand post-traumatic stress disorder and its treatment.* Western Sydney University (Australia);2017.

[43] UNHCR: UNHCR in Pakistan: An enduring partnership. 2020. Accessed April 2020. Reference Source

[44] UNHCR: *Afghan Refugees Camp Population in KP March, 2018.* Brussels: United Nations High Commissioner for Refugees;2018.

[45] Commissionerate for Afghan Refugees. Punjab Figures at a Glance.Accessed April 2020. Reference Source

[46] Vahedi S : World Health Organization Quality-of-Life Scale (WHOQOL-BREF): Analyses of Their Item Response Theory Properties Based on the Graded Responses Model. *Iran J Psychiatry.* 2010;5:140–153. 22952508 PMC3395923

[47] Topp CW Ostergaard SD Sondergaard S : The WHO-5 Well-Being Index: a systematic review of the literature. *Psychother Psychosom.* 2015;84:167–176. 10.1159/000376585 25831962

[48] Hollifield M Verbillis-Kolp S Farmer B : The Refugee Health Screener-15 (RHS-15): development and validation of an instrument for anxiety, depression, and PTSD in refugees. *Gen Hosp Psychiatry.* 2013;35:202–209. 10.1016/j.genhosppsych.2012.12.002 23347455

[49] Chinn D McCarthy C : All Aspects of Health Literacy Scale (AAHLS): developing a tool to measure functional, communicative and critical health literacy in primary healthcare settings. *Patient Educ Couns.* 2013;90:247–253. 10.1016/j.pec.2012.10.019 23206659

[50] Doocy S Lyles E Akhu-Zaheya L : Health service access and utilization among Syrian refugees in Jordan. *Int J Equity Health.* 2016;15:108. 10.1186/s12939-016-0399-4 27418336 PMC4946096

[51] Doocy S Lyles E Akhu-Zaheya L : Health Service Utilization among Syrian Refugees with Chronic Health Conditions in Jordan. *PLoS One.* 2016;11:e0150088. 10.1371/journal.pone.0150088 27073930 PMC4830531

[52] Doocy S Lyles E Roberton T : Prevalence and care-seeking for chronic diseases among Syrian refugees in Jordan. *BMC Public Health.* 2015;15:1097. 10.1186/s12889-015-2429-3 26521231 PMC4628338

[53] Dembe AE Partridge JS Geist LC : Statistical software applications used in health services research: analysis of published studies in the U.S. *BMC Health Serv Res.* 2011;11:252. 10.1186/1472-6963-11-252 21977990 PMC3205033

[54] Malik MS Afzal M Farid A : Disease Status of Afghan Refugees and Migrants in Pakistan. *Front Public Health.* 2019;7:185. 10.3389/fpubh.2019.00185 31334212 PMC6616124

[55] UNHCR: *Report on Issues faced by Afghan Refugees during Repatriation.* Brussels: United Nations High Commissioner for Refugees;2012.

[56] Tribune TE : Fighting Disease: Gilani blames refugees for polio spread. *Bilal Ali Lashri.* 2011.

