# Supplementary material for: Protocol for a cross-sectional study on factors affecting health-related quality of life among Afghan refugees in Pakistan
Source: F1000Res. 2024 Oct 14;10:971. Originally published 2021 Sep 27. [Version 2] doi: 10.12688/f1000research.73005.2 (PMC11140308; doi:10.12688/f1000research.73005.2)
Supplement: Peer review history

# Reviewer report: Reviewer response for version 1

**DOI:** 10.5256/f1000research.76623.r275681

**Published:** 2024-05-30

**Authors:** Izaddin Ahmad Aziz

**Affiliations:** 1 special education, Salahaddin University - Erbil, Erbil, Kurdistan Region, 0044, Iraq

In general, provides a clear and comprehensive overview of the study's objectives and methodology. The introduction effectively highlights the importance of addressing the factors that impact the health-related quality of life of Afghan refugees in Pakistan, setting a strong foundation for the research. The logical flow of information and the detailed explanation of the study design and methods demonstrate thorough planning and preparation for the study. Overall, the introduction is well-written and engaging, effectively capturing the reader's interest and setting the stage for the rest of the research.

 The good approach to data collection and analysis, including the use of standardized instruments and statistical software, is commendable and will likely yield reliable and valid results. The inclusion of key variables such as socio-demographic characteristics, health status, and socio-economic factors will provide a holistic understanding of the factors influencing health-related quality of life among Afghan refugees in Pakistan.

Is the study design appropriate for the research question?

Yes

Is the rationale for, and objectives of, the study clearly described?

Yes

Are sufficient details of the methods provided to allow replication by others?

Yes

Are the datasets clearly presented in a useable and accessible format?

Partly

Reviewer Expertise:

Psychology, imigration issues

I confirm that I have read this submission and believe that I have an appropriate level of expertise to confirm that it is of an acceptable scientific standard.

# Response

**Published:** 2024-10-05

**Authors:** Florian Fischer

**Affiliations:** Charité-Universitätsmedizin Berlin, Germany

Thank you very much for this positive feedback.

# Reviewer report: Reviewer response for version 1

**DOI:** 10.5256/f1000research.76623.r148759

**Published:** 2024-05-25

**Authors:** Shraddha Kashyap

**Affiliations:** 1 University of Western Australia, Perth, Australia

Thank you for the opportunity to review this protocol, which describes the methodology of a study aiming to assess the health status (including wellbeing, mental health, health literacy, and factors affecting health and health care) among Afghan people with refugee backgrounds, living in Pakistan.

 This paper describes rigorous sampling techniques in a complex environment, and it is clear and well written. It is an important area of work.

- I note that the study (for which this is a protocol) has already been conducted. Could authors provide a justification for publishing the methodology as a separate protocol rather than publishing the methods together with results, and a discussion of the implications of the findings?
- The cross-sectional nature of the design is limiting in that causal associations between environmental factors and wellbeing cannot be made, however, the results will provide helpful information as a snapshot of the health status of Afghan refugees living in Pakistan.

- Given the time frame in which data was collected (March 2020-May 2021), can authors clarify whether there were any measures directly related to the impacts of COVID on participants’ wellbeing?

The following queries for authors are more conceptual and are suggestions to consider for this and/or future studies.

 Authors have acknowledged that factors such as literacy levels have led to certain groups of people being excluded from the study, and this is understandable given that the study requires participants to complete written surveys.  At the same time, is it possible that the people excluded from the study are the most vulnerable, and find themselves frequently excluded from such efforts to provide appropriate support?

 As a quantitatively trained researcher myself who has worked on similar projects using similar methodologies, my question is as follows; is our understanding of factors such as wellbeing, mental health, and health among refugee populations limited by how we have chosen to measure them?  That is, by only using quantitative methodologies, which requires participants to complete written surveys as the only option.

 Could authors discuss whether or not they considered collecting qualitative data, to complement the quantitative findings (e.g., by collecting data relating to narratives about wellbeing, told by people who may not be able to complete surveys)?  This would account for all literacy levels, potentially enhance the cultural sensitivity of the project, and improve the validity of findings.

- If this was considered, could authors describe any barriers faced in doing this?

Further, despite rigorous forward and back translation of surveys, can factors as culturally and contextually bound such as concepts of mental health, distress, quality of life, and wellbeing, be **accurately and fully** captured for diverse Afghan communities, from adaptations of measures designed from a Western paradigm of mental health and wellbeing? The following are examples of papers which discuss this issue:

 1) Culture and reform of mental health care in central and eastern Europe. WHO, (2017 ^1^)

 2) Carpenter-Song et al. (2010 ^2^)

 3) Parker H. et al. (2003 ^3^)

- Could authors provide some reflections on this?

Is the study design appropriate for the research question?

Partly

Is the rationale for, and objectives of, the study clearly described?

Yes

Are sufficient details of the methods provided to allow replication by others?

Yes

Are the datasets clearly presented in a useable and accessible format?

Not applicable

Reviewer Expertise:

Mental health

I confirm that I have read this submission and believe that I have an appropriate level of expertise to confirm that it is of an acceptable scientific standard, however I have significant reservations, as outlined above.

## References

[1] Culture and reform of mental health care in central and eastern Europe. *WHO* .2018; Reference source

[2] : Ethno-cultural variations in the experience and meaning of mental illness and treatment: implications for access and utilization. *Transcult Psychiatry* .2010;47(2) : 10.1177/1363461510368906 224-51 10.1177/1363461510368906 20603387

[3] : Schizophrenia and Related Psychosis in Aboriginal and Torres Strait Islander People. *Aboriginal and Islander Health Worker Journal* .2003;27(5) :17-19 Reference source

# Response

**Published:** 2024-10-05

**Authors:** Florian Fischer

**Affiliations:** Charité-Universitätsmedizin Berlin, Germany

Registering the research protocol at the national level helps establish project novelty and priority. This is crucial as similar studies might be initiated simultaneously at different institutions. If another study on the same topic and population is published before ours, protocol registration can demonstrate our research's originality and timeline. This process ensures that our project's unique contributions are recognized despite overlapping research.

 Research article on Health related quality of life of Afghan Refugees was published before our detailed article from Pakistan.

 The survey adopted a quantitative research method because it provides the advantages of large sample size, more accurate, rapid numerical data collection, presentation of collected data in the form of graphical display, and results interpretation to generate useful information for decision-making (Conroy et al., 2008). The cross-sectional survey design was selected because it allowed researchers to instantly gather information from a large number of respondents at one point in time and compare group variations in a time-efficient and economical way (Kesmodel, 2018). This cross-sectional investigation only gives a snapshot of limited factors affecting the health-associated quality of life among Afghan refugees in a precise time and environment and the number and nature of determinants may vary over the period. A standard causal relationship between sociodemographics characteristics, predictors, and the outcome variable could not be established due to the cross-sectional design of the current investigation. The longitudinal investigation was not planned due to the time and financial limits.

 The fact that this research relies on refugee interviewed /self-documented data during the COVID-19 pandemic, which might not fully reflect real behavior, was also one of the survey's main limitations.

 The survey was executed by a team of two principal researchers and four bilingual Afghan refugee research associates who assisted the sampled Afghan refugees in filling out the questionnaires on paper and performed in-person interviews with respondents who were illiterate or requested interviews. Only refugees who were unable to understand consent procedures or respond to a survey questionnaire due to cultural hesitancy were not included in the investigation.

 A survey on Afghan refugees' health care in Pakistan was challenging to conduct due to a diverse range of issues. The individuals contacted by organizations working with refugees declined to share refugee lists with contact numbers because of the non-data sharing policy about refugees due to security and confidentiality concerns. Instead, we used online statistics about refugees in Pakistan to approximate about population in each area and contacted the locals in charge of refugee regions to assist in surveys. Afghan refugee research associates were reluctant to reveal their identity at any stage of the project due to unknown security concerns. Hiring bilingual Afghan refugees and traveling to these areas to meet locals in charge of survey permission and fieldwork were big challenges. It took almost 18 months to complete the desired sample size with high nonresponsive rates in these areas.

 The response rate for the current investigation was 55.45 %. The 2137 respondents were approached to complete the sample size of 1185 participants. The population was quite sensitive and emotional if they did not like any question, facial expression, and clothing of the investigator, conversation with the female investigator, or use of mobile they abruptly abandoned the questionnaire or interview before it was even halfway completed. Our team members adhere to the local dress code and ethical guidelines and stay away from everything that would interfere with the research study. In the majority of the districts, only one female was permitted to approach a female at home for the research, and men were more likely to give interviews outside of their homes, in camps, or tea shops.

 Qualitative data complement quantitative results by providing deeper insights into the subject matter. This combination enhances the validity of findings and offers a more comprehensive understanding of the research topic. Qualitative data collection was initially planned but University DPCC based on the conceptual framework only permitted the Quantitative Survey.

# Reviewer report: Reviewer response for version 1

**DOI:** 10.5256/f1000research.76623.r130109

**Published:** 2022-04-27

**Authors:** Alejandro Gil-Salmerón

**Affiliations:** 1 International Foundation for Integrated Care, Oxford, UK

I would like to congratulate all of the authors on this nicely presented protocol.

 My only concern is that the begining of the abstract starts addressing "accessibility to health care", and there are studies focused on this aspect of refugees' health and social determinants of health, whereas the protocol addresses "health-related quality of life" as the main varaible, and access to healthcare services is the "dependent variable", so the authors should explain how health-related quality of life could be affected by accessibility. 

 This might create some discordance with the rest of the text.

 I would suggest the authors include some of the results of a large-scale project in Europe. The first one is a scoping literature review, the second one an analysis of the health status and needs, and the third one is an analyis of the accessibility by percieived discrimination in healthcare settings and avaiability of transaltion services:

- Lebano *et al. *(2020 ^1^).
- Riza *et al.* (2020 ^2^).
- Gil-Salmerón *et al.* (2021 ^3^).

Is the study design appropriate for the research question?

Yes

Is the rationale for, and objectives of, the study clearly described?

Yes

Are sufficient details of the methods provided to allow replication by others?

Yes

Are the datasets clearly presented in a useable and accessible format?

Not applicable

Reviewer Expertise:

integrated care; health services research; social and preventive medicine; social determinants of health

I confirm that I have read this submission and believe that I have an appropriate level of expertise to confirm that it is of an acceptable scientific standard, however I have significant reservations, as outlined above.

## References

[1] : Migrants' and refugees' health status and healthcare in Europe: a scoping literature review. *BMC Public Health* .2020;20(1) : 10.1186/s12889-020-08749-8 1039 10.1186/s12889-020-08749-8 32605605 PMC7329528

[2] : Determinants of Refugee and Migrant Health Status in 10 European Countries: The Mig-HealthCare Project. *International Journal of Environmental Research and Public Health* .2020;17(17) : 10.3390/ijerph17176353 10.3390/ijerph17176353 PMC750373532878303

[3] : Access to Healthcare for Migrant Patients in Europe: Healthcare Discrimination and Translation Services. *International Journal of Environmental Research and Public Health* .2021;18(15) : 10.3390/ijerph18157901 10.3390/ijerph18157901 PMC834533834360197

# Response

**Published:** 2024-10-05

**Authors:** Florian Fischer

**Affiliations:** Charité-Universitätsmedizin Berlin, Germany

The fact that a certain number of the predictor variables in surveys weren't always independent or dependent must be kept in mind. A predictor variable that was independent for one survey could become a dependent variable in another depending on the research survey (Flannelly et al., 2014). This assumption should be kept in mind while analyzing the survey's results because predictors were utilized in previous surveys as both independent and dependent variables, such as self-rated health (Vogel et al., 2021; Williams et al., 2017), substance abuse(smoking, naswar) (Abernathy et al., 1966), cultural compatibility (Iivari & Iivari, 2011), linguistic barriers, face discrimination (Salleh et al., 2021), social inclusion (Nihinlola, 2020), access to clean water, access to sanitation (Purba & Budiono, 2019), access to education, access to healthcare information via social, print or electronic media, access to healthcare, chronic health illness (Grønning et al., 2018), last time visited health facility, socioeconomic support from government or donor agencies (Nihinlola, 2020), health care cost affordability. Confounders were adjusted at the design stage with demographic randomization of the sample in all the selected regions. Age group, gender, and health illness quota matching of all the selected strata was done to minimize the confounder effect. Multivariate statistical models were applied to control the confounders at the analysis stage.

 Health-related quality of life (HRQoL) encompasses various aspects of an individual's overall well-being, including physical, mental, and social health. Accessibility plays a crucial role in determining HRQoL, as it directly influences how individuals can interact with their environment and access necessary resources and services. Accessibility impacts health-related quality of life (HRQoL) by affecting physical, economic, social, and psychological aspects. Limited physical access to environments and services can hinder mobility and independence, while economic barriers can restrict access to healthcare and employment. Social accessibility affects inclusion and participation, while inadequate communication access can lead to isolation. Improving accessibility helps enhance HRQoL by ensuring individuals can fully engage in daily activities and maintain overall well-being. All the domains are discussed in detail in final result as mentioned in link below: https://www.researchsquare.com/article/rs-3925465/v1

 As per suggestion relevant literature was added as mentioned in table 1.

 The literature from developed countries, including the European nations, United States, and Australia focuses on various aspects of refugee health assessment, such as health-related quality of life, mental health screening, and access to healthcare, food insecurity, and language proficiency in the host country. However, the specific type of health assessment conducted may vary depending on the country's policies and the author's research focus.
